# Prognostic value of different immunohistopathological patterns and Ki67 proliferation index for Merkel cell carcinoma

**DOI:** 10.1186/s13005-025-00578-7

**Published:** 2025-12-23

**Authors:** Katharina Theresa Obermeier, Wenko Smolka, Maximilian Kollmuss, Sabina Noreen Wuersching, Ina Dewenter, Philipp Poxleiter, Sven Otto, Yoana Malenova, Florian Fegg, Paris Liokatis

**Affiliations:** 1 Department of Oral and Maxillofacial Surgery and Facial Plastic Surgery, University Hospital, LMU Munich, Lindwurmstrasse 2a, Munich, 80337 Germany; 2 Department of Conservative Dentistry and Periodontology, University Hospital, LMU Munich, Goethestrasse 70, Munich, 80336 Germany

**Keywords:** Merkel cell carcinoma, Ki67-index, Prognostic marker, Neuroendocrine skin tumors, Lymphatic metastasis

## Abstract

Merkel cell carcinoma (MCC) is a rare and aggressive neuroendocrine skin tumor, which preferentially appears in the head and neck area. Overall 5-year survival is about 66–70% in patients with non-metastasized tumors and reduces to 42% in cases with regional metastases, and down to 18% in case of distant metastases. The Ki67- index is one of the most important biological markers in neuroendocrine tumors and is recently known as a prognostic marker in pancreatic neuroendocrine neoplasms. This study aims to evaluate the prognostic role of Ki67 in Merkel cell carcinoma. A cut-off value for Ki67 index was calculated using Youden’s index. Overall, 16 patients were included in this retrospective study. The best matching cutoff value for our data was calculated at 77.5%. Ki67 turned out to be > 85% in patients with local recurrence of MCC and > 90% with lymph node metastases. A Ki67-index of 77.5% stratified the patients in our cohort most efficiently. A safety margin higher than 1 cm should be aimed.

## Simple summary

Merkel cell carcinoma is a rare aggressive neuroendocrine skin tumor. General 5-year survival is between 66-70% in patients without metastases. This study aims to present the prognostic role of Ki67 proliferation index in Merkel cell carcinoma. This study shows that a high Ki67-proliferation index is linked to a higher risk for metastases and recurrence. In this study, it was also shown that the elevated Ki67 index was associated with poorer 5-year survival.

## Introduction

Merkel cell carcinoma (MCC) is a rare but aggressive neuroendocrine tumor of the skin, which has increased in prevalence over the past years [1]. Head and neck are the preferred localization of MCC [2]. Chronic sun exposure, age, male gender, immunosuppression (HIV infection, immunosuppressive therapy due to organ transplantation or autoimmune diseases), and fair skin predispose to developing MCC [3, 4]. Typically, MCC presents as an erythematous node or plaque-like skin lesion [5]. However, due to the wide histopathological patterns the differential diagnosis of malignant small blue cell tumor is often difficult. In addition local and systemic progression is often rapid [6]. Regional lymph node metastases are the most common metastases in MCC. Early state MCC can be treated curatively with tumor resection and eventually sentinel lymph node biopsy or neck dissection [7]. Early tumor stages I and II are characterized in the TNM classification by a tumor diameter smaller or larger than 2 cm, respectively, with otherwise unremarkable lymph node status. All later tumor stages are characterized by the presence of lymph node or distant metastases alone, regardless of diameter. For an extensive disease with late diagnosis, multidisciplinary therapy options combining surgical tumor resection, chemoradiotherapy, and immunotherapy should be implemented [8].

The prognosis of MCC varies according to tumor size and the presence of metastases. The five-year survival rate for a non-metastasized tumor smaller than 2 cm is 66–70%. If tumor spreading is present, the survival rate decreases to 42–52% for lymph node metastases and 18% for distant metastases [9–11]. Further potential risk factors for a worse prognosis are lymphovascular invasion, p53, p63 and CD34 immunopositivity [12, 13].

Among the parameters studied to classify patients with MCC are the presence of the Merkel cell polyomavirus (MCPv) and the Ki67-index. The MCPv was first described in 2008 and is found in more than 90% of all MCC. It is an oncogenic, uncoated double-stranded DNA-Virus. The MCPv is found in more than 90% of patients not suffering from Merkel cell carcinoma and its role in MCC is controversial. Regarding the MCPv, there is probably no significant association with survival or response to treatment [14, 15], although it plays a role in the carcinogenesis of most tumors and is initially thought to be associated with a better prognosis [16].

Ki-67 was first identified as an antigen in Hodgkin lymphoma cell nuclei [17]. Since 1983 Ki67 became one of the most important antigens in neoplasm [18]. The Ki67- index is one of the most important biological markers in neuroendocrine tumors [18] and is recently known as a prognostic marker in pancreatic neuroendocrine neoplasms [18] and other neuroendocrine tumors [19]. In the update from the 5th Edition of the world health organization classification of head and neck tumors concerning immunohistology, Klöppel et al. 2020 described the Ki67 proliferation index as an important factor in MCC prognosis and clinical behavior. The Ki67 proliferation marker is primarily used to diagnose neuroendocrine tumors as it labels the nuclei of proliferating non-neoplastic and neoplastic cells and could assess the proliferative activity of the tumor [19]. In 2000, the Ki67 index was included in the WHO classification of gastroenteropancreatic neuroendocrine neoplasms [20]. A pending issue is the definition of a cutoff for tumors with higher recurrence risk. For gastrointestinal neuroendocrine carcinomas, a threshold of 55% is associated with aggressive tumors. This cutoff is still to be validated for MCC and merely for MCC in the head and neck, which could be associated with more aggressive behavior than MCC in other areas [21].

The aim of this study is to give an overview of 16 patients with MCC treated in our hospital. Emphasis is given to the Ki67 proliferation index as a parameter for patient stratification. Moreover, other clinical, histological and immunohistological parameters and their possible impact on lymph nodal metastases, distant metastases, local recurrence, and overall survival were analyzed.

## Materials and methods

**Fig. 1:**
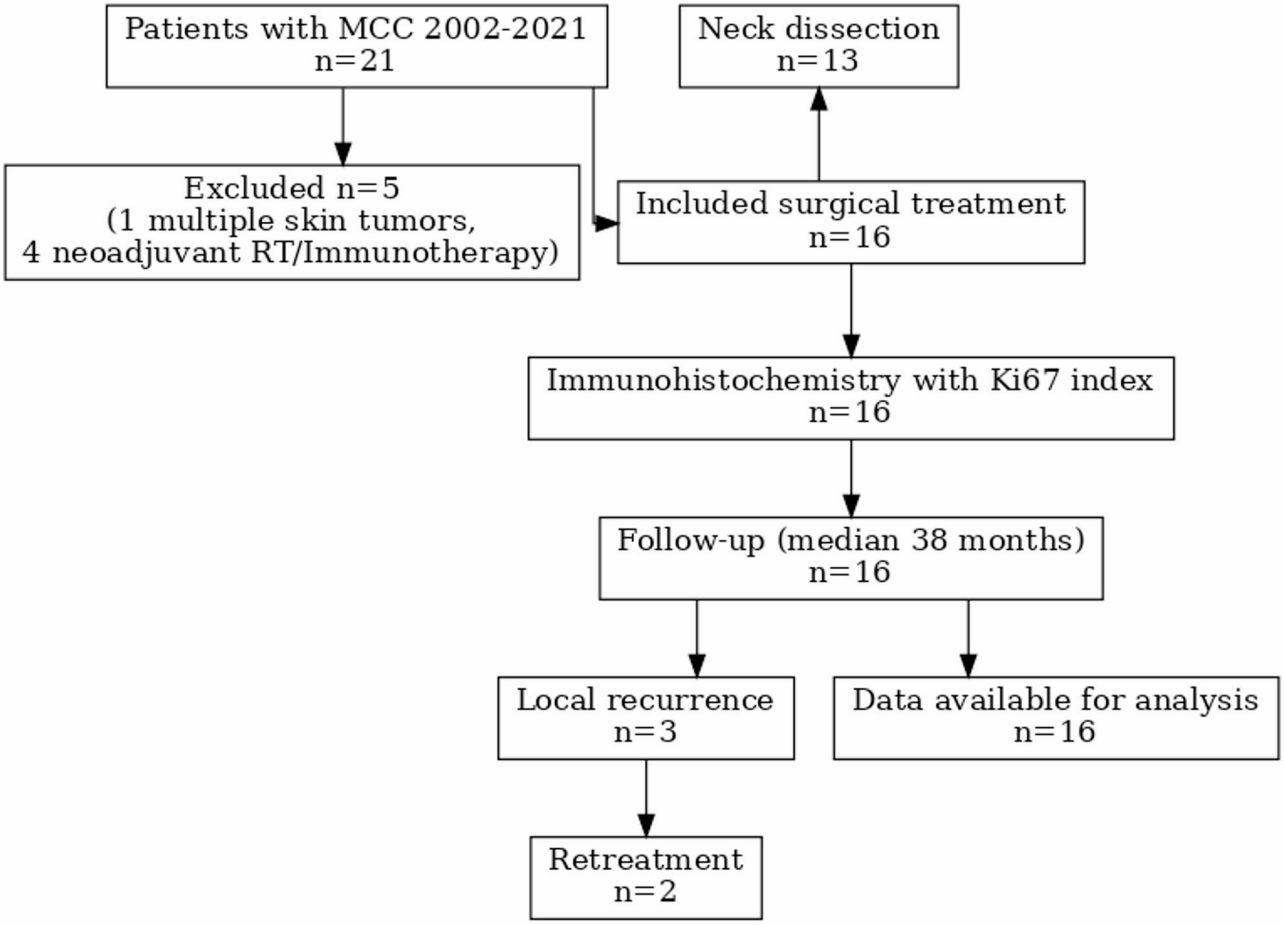
Data curation represented in a flowchart

This retrospective study was approved by the institutional review board of the University Hospital of Munich, Germany (Munich, Germany; UE Nr 20-1096). Informed consent was waived by the institutional review board of the University Hospital of the Ludwig-Maximilians-University Munich, Germany due to the retrospective nature of the data. All research was performed in accordance with the guidelines of the Declaration of Helsinki. Inclusion criteria were primary diagnosis of a MCC in the head and neck and absence of a history of head and neck cancer. Exclusion criteria included a history of previous irradiation in the head and neck area or neck dissection, patients who underwent chemo- or immunotherapy or patients who were immunosuppressed. The minimum follow-up in our study was two years. Figure 1 gives an overview of data curation and patients.

The demographic, oncological and treatment data were obtained via the medical records, radiographics and pathological reports. If specific pathological data were missing in an early case, they were examined retrospectively. Patients´ medical records have been screened, evaluated and reported according to the STROBE (STrengthening the Reporting of OBservational studies in Epidemiology) guidelines. All methods were performed in accordance with the guidelines and regulations of this journal.

### Evaluation of the Ki67-index

Ki-67 indices were retrieved from histopathological assessments conducted within the framework of routine diagnostic procedures. When evaluating the pathological data all reports of Ki67-index were already available.

### Evaluation of margins

The margins were calculated by adding the safety margins on the tumor preparation and the frozen sections. Both were added and given in mm in pathological report. All margins were examined by an experienced pathologist.

### Statistical analysis

Statistical analysis was conducted using SPSS^®^ 24 version 4.0 (SPSS Inc., Chicago, IL, USA). Due to the small number of patients, no statistical test was used, and the data were primarily qualitatively and descriptively analyzed. The optimal cut-off for the Ki67 index for the stratification of the patients regarding survival was investigated using Youden’s Index for the receiver operating characteristic (ROC) curve analysis. Survival analysis was performed by using Kaplan-Meier Analysis and was performed in Python 3.8.0 using the packages numpy, lifelines and matplotlib.

## Results

### Demographic data

**Fig. 2:**
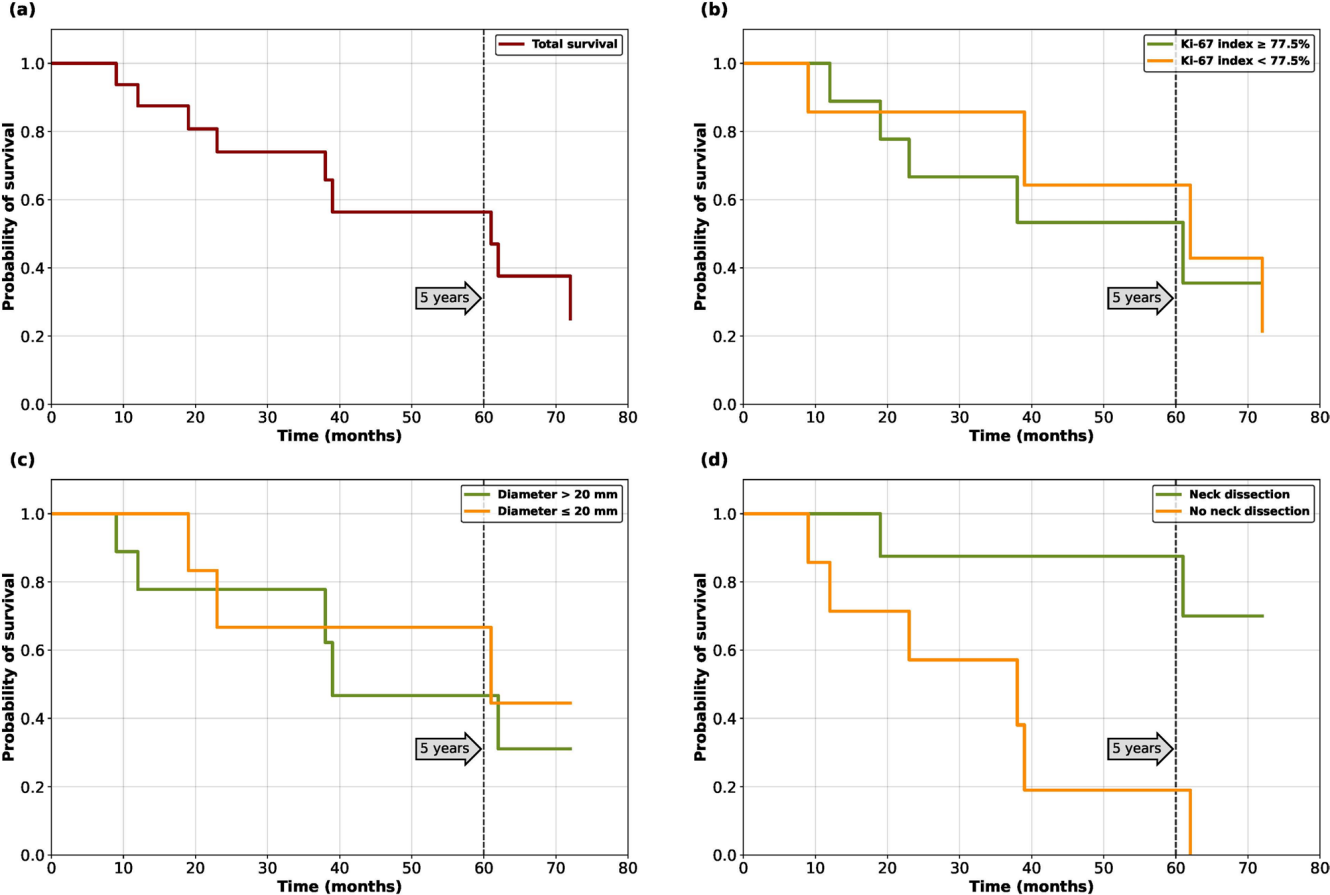
Survival curves. **a **total survival. **b **survival considering Ki67. **c **Survival considering diameter. **d **Survival considering neck dissection

Sixteen (16) patients treated in our hospital between 2002 and 2021 were included in the retrospective study. Twelve (12) patients were female, and four (4) were male. One of the patients had dementia, and two of the patients had a severe cardiac history. One of the patients had additional multiple basal cell carcinomas and cutaneous squamous cell carcinomas combined with MCC. The median age was 81.5 years, ranging from 65 to 93 years. All patients were Caucasians with phototypes I and II. In our cases series, the MCC appeared in three different localisations in the head area: in 12 patients, it was located on the cheek, in 3 patients on the upper eyelid, and in one on the ear. All patients underwent computed tomography (CT) for staging. None of the patients had received immunosuppressive therapy before, and all patients were treated primarily with a resection without a neoadjuvant protocol. Overall 10 patients (62.5%) died during follow-up time. All patients died due to the tumor disease or a consequence thereof. In four cases due to lymph node metastases in the neck and in 6 cases due to distant metastases. The overall 5-year survival probability was slightly more than 60% in this cohort. No censored observations occurred in this patient cohort. Survival analysis is shown in Fig. 2.

### Oncological data

All patients in this study had a primary diagnosis of MCC. In all patients, the p16-status was evaluated and was negative in all patients. The average diameter of the primary tumors was 23.5 mm (2–46 mm), and the average thickness of infiltration was 7.5 mm (2–22 mm). The mean safety margins were 12.1 mm (st.d. 4.8, range 5–21 mm). The mean overall survival was 44 months (st.d. 18, range 9–96).

All patients underwent surgical treatment meaning tumor resection, with nine patients receiving a selective neck dissection as further treatment due to suspect lymph nodes in the staging examinations. In five cases, neck dissection was performed on the ipsilateral side and in four cases, on both sides. No patients received a sentinel node biopsy, alone. Due to the extent of the primary tumor, three patients underwent a microvascular reconstruction of the cheek, while in the rest, a regional flap was used to reconstruct the defect. As microvascular flap latissimus dorsi flap was used in one case and in two cases radial forearm-flap. One patient (patient 14) received an adjuvant radiotherapy. An overview of oncological data is shown in Table 1. Survival Curves of patients are shown in Fig. 2 (a, b, c, d). Survival curves show that patients with high Ki67-proliferation index have worse survival, as well as patients with higher tumor diameter. Our data shows, that patients with neck dissection have a better 5- year survival rate compared to the ones without neck dissection.

**Table 1:** Oncological data

Patient	Age	Gender	TNM	Localisation	Neck Dissection	Adjuvant treatment	Local Recurrence	Diameter	DOI	Ki67	Survival (months)	Follow-up time after surgery	Minimum margin (mm)	Treatment after recurrence
1	77	Fem.	T1NxMx	Cheek	-		0	30	11	90%	38		15	
2	92	Fem.	T2NxMx	Cheek	-		0	24	7	90%	12		8	
3	73	Male	T2N1Mx	Cheek	I-III ipsi.		0	19	8	90%	96		5	
4	89	Fem.	T1NXMx	Upper lid	-		0	2	12	90%	23		20	
5	83	Fem.	T1N0Mx	Cheek	I-III ipsi.		1	11	4	85%	61		10	Surgery 4 cycles Etoposid, Taxol, Carboplatin, Radiotherapy
6	87	Fem.	T4N0Mx	Upper lid	I-III ipsi.		0	29	18	70%		84	8	
7	84	Fem.	T2N0Mx	Cheek	I-III b.s.		1	14	7	90%	19		8	Surgery
8	93	Male	T2NxMx	Cheek	-		0	36	13	60%	62		21	
9	73	Fem.	T1NxMx	Upper lid	-		0	25	9	25%	9		15	
10	65	Fem.	T1N0Mx	Cheek	I-V b.s.		1	19	10	85%		38	9	No treatment
11	81	Fem.	T4aNxmx	Cheek	-		0	30	5	25%		24	10	
12	89	Fem.	T2NxMx	Cheek	-		0	23	6	40%	39		15	
13	80	Fem.	T2N1Mx	Cheek	I-III ipsi.		0	46	22	20%	86		19	
14	81	Male	T2N1Mx	Ear	I-IV b.s.	Radiotherapy	0	30	2	90%		36	9	
15	82	Male	T4N1M1	Cheek	I-III b.s.		0	11	5	90%		63	12	Avelumab
16	65	Fem.	T1N0Mx	cheek	I-III ipsi.		0	7	2	25%		13	10	

### Immunohistochemistry and Ki67 index

All tumors showed similar histopathological characteristics: small to intermediate cells, monomorphic nuclei, dispersed chromatin and expression of CK 20 and chromogranin A was found in 15/16 specimens. CD 56 expression was found in 5/16 cases, CD79a in 1/16 cases, CD20 in 3/16 cases, CD3 in 3/16 cases, synaptophysin in 8/16 cases and pan-keratin in 4/16 cases.

**Fig. 3:**
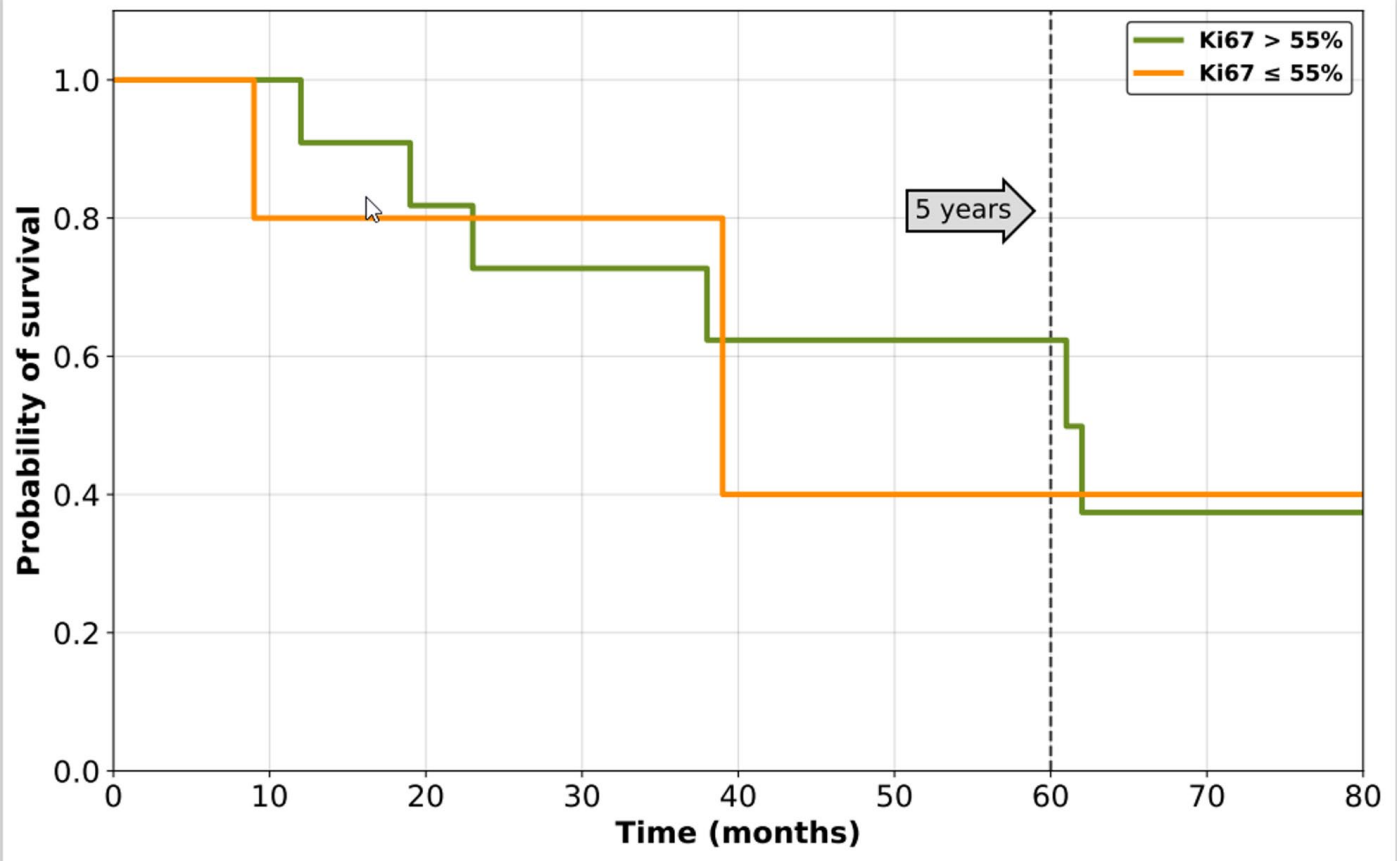
Survival Curve of Ki67-index 55%

The mean Ki67- index was 67%. We calculated the Youden’s Index using the ROC curve to detect the ideal cutoff value. The best matching cutoff value for our data was calculated at 77.5%. For a comparison with the cut-off of 55% from the guideline, a survival curve was calculated (Fig. 3).

### Patients with local recurrence

Overall three patients (patients 5, 7, 10) suffered from a local recurrence of MCC. The safety margins were 10 mm, 8 mm and 9 mm. Local recurrence appeared after 7, 4 and 6 months, respectively. The Ki67- index for these patients was 85% 90% and 85%, respectively. In patients 5 and 7 the occurrence was treated surgically. In patient 5 an additional postoperative chemoradiotherapy with 4 cycles Etoposid, Taxol, Carboplatin was performed, too. Patient 10 rejected any further treatment. The overall survival was 23, 19 and 38 months, respectively.

### Patients with lymph node metastases and distant metastases

Four of the nine patients (patients 3, 13, 14, 15) who received a neck dissection due to suspected lymph nodes were positive for lymph node metastases. Patient 15 also suffered from distant metastasis in the lung. Three of these patients (3, 14, 15) had a Ki67-index of 90%, and one (patient 13) had a Ki67- index of 20%. The average survival was 70 months (st.d. 27, 36–96). The recurrence in the lung in patient 15 was treated with an immunotherapy with avelumab leading to a remission of the disease for nearly 50 months after the appearance of the lung metastasis. Overall MCC with Ki67 > 77.5% were more frequently observed in patients with progressive/aggressive disease, local recurrence and lymph node metastases and distant metastases than in patients free of disease.

## Discussion

MCC is a rare, aggressive neuroendocrine tumor affecting, on average, older patients with variable prognosis, similar to our cohort [22]. Because of the rarity of the disease, most studies have included only few patients and data is generally limited. This problem becomes more pronounced if we concentrate on the head and neck area, where specific questions are not adequately addressed. The risk factors of lymph node metastases and, therefore, the necessity of neck dissection is often a question to be answered for each patient. The guidelines suggest performing at least a sentinel node biopsy in MCC due to possible occult metastases not detected by staging examinations. However, the older age of patients suffering from MCC compared to other cancers and the existing comorbidities may raise essential restrictions on performing extended surgical treatments. The high number of deaths (62.5%) is certainly also due to the high age of the patients and the associated morbidities as well as due to tumor associated complications such as pulmonary embolism or the side effects of chemotherapy.

Moreover, a safety resection margin of 1–2 cm is suggested. However, in most cases of MCC in the head and neck region, this is not feasible without damaging important organs or causing severe defects requiring extended reconstruction. Identifying risk factors for the local aggressivity of the tumor could offer an individualized approach concerning local resection, neck dissection or adjuvant treatment. In our study 6 patients (37.5%) had margins < 1 cm, although 1–2 cm is recommended. In all patients the basal margin was < 1 cm. As already mentioned, tumor resection in the head and neck region is a challenge for the surgeon. The proximity to the skull base or to large neck vessels was the limiting factor here and prevented a more extensive resection. Extensive resections lead to nerve damage and ultimately to functional losses with regard to chewing problems, dysphagia or swallowing function. The facial mimicry can also be impaired by damage to the facial nerve. A drooping mouth-angle or an insufficient lid closure can negatively affect the quality of life of patients [23].

In our study, the 5-year overall survival was 60%, which is slightly lower than in the literature, likely owing to the older average age (81 years old) in comparison to similar studies [24] and the severity of the cases referred to a university clinic. Kaplan-Meier analysis shows survival considering Ki67-proliferation index. As shown in Fig. 2 survival in patients with Ki67 higher than 85% is worse compared to patients with a lower index. In the Kaplan-Meier analysis, of course, the small patient cohort has to be considered critically, but on the other hand, the numbers are similar to numbers from other studies. Patients with greater tumor diameter had a worse survival compared to smaller diameters, which is similar to other studies [25, 26].

Due to the low number of studies, especially if we focus on the head and neck, the role of Ki67 on patients’ prognosis is very heterogeneous. Some studies report an association between a high Ki67- index and more aggressive growth of the tumor and worse outcomes [27, 28]. There are cut-off values decribed by Sorbye et al. 2013 and Milione et al. 2017 (Ki67 < 55% and Ki67 > 55%) for neuroendocrine carcinomas. They came to the conclusion that tumor morphology as well as Ki67 could be useful as prognostic factors and be useful for tumor classification [29].

The cut-off value suggested in the current study differs significantly from the 55% proposed before, which originates from the findings on digestive neuroendocrine carcinomas. We also used Youden index analysis which identified a Ki67 value of 77.5% as the best cut-off. In our study the cut-off value was higher than in other studies, but mean Ki67 proliferation index was also higher. La Rosa et al. 2020 had a mean Ki67 proliferation index of 51.3% and they defined their cut-off with 55% [30]. Compared to our study mean Ki67 index was 67% and Youden index analysis and ROC curve defined 77.5% to be the best matching cut-off for our data. Unfortunately, due to the lack of data, defining a cut-off especially for patients with MCC in the head and neck is still not possible. The findings of this study indicate that the general proposed cut-off of 55% may not always apply. Another possibility for a clinically practical patient stratification could be the definition of two cut-offs, to identify patients with very low or very high Ki67 indexes and recurrence risk. The Ki67-index of 77.5% was the best statistical fitting value for our data of the 16 patients. However, in clinical practice, this value is difficult to calculate and to implement in the evaluation. With the eye-balling method described above, only approximate values can be given. The cutoff-value of 77.5% is solely a statistic value, indicating the risk for metastasis and recurrence. Regardless, this value is a first impression of a quite small sample size and should not implement a fixed guideline. Perhaps, in the future, more precise methods, which are just not as time-consuming in everyday practice, can be found to facilitate this. In general, one can therefore say that the cut-off value of 77.5% is only a statistically calculated value, which is intended to function as a approximate guide in the prognosis assessment. In literature the 55% cut-off value is described. Figure 3 shows that the 5-year survival in patients with Ki67-index > 55% is better than in our calculated cut-off of 77.5%. On the other hand only 5 patients had a Ki-67-index lower than 55% and by statistical analysis the most fitting cut-off for our data was 77.5%. In order to find a more accurate cut-off, a much larger cohort of patients should be studied. Although this study has only a low number of patients included, slightly tendency for the prognosis of MCC with a Ki67-index could have been shown.

Four of the patients with lymph node metastases were staged T2 only one patient already had an advanced stage (T4a). This patient developed a lung metastasis a few months after the operation. Interestingly, a long remission was achieved after the immunotherapy treatment with avelumab, which is in consistency with existing data [31, 32]. Generally, immunotherapy is gaining importance in the treatment of MCC and it may be the vehicle for an individualised treatment of MCC [24].

Due to the small number of patients in this study, it cannot be concluded if a Ki67 index higher than 77.5% is indicative of a higher risk for node metastases. Only slightly tendence for worse survival can be shown. Although three of the four patients with metastasized lymph nodes had an index of 90%, the forth had an index of 25%. Additionally, there were three patients with an index > 85% with no findings in the neck dissection. Neck dissection in MCC is discussed controversially in the literature. Interestingly in our study 5-year-survival in our patients is higher in patients with neck dissection.

According to our data, T-stage rather than Ki67 could be the primary predictor regarding regional metastases. A pN1-patient had a Ki67-index of 20% but a tumor diameter of 46 mm and a depth of 22 mm. Although there are only 16 patients included in this study, our survival curve shows, that a larger tumor-diameter (> 2 cm diameter) has negative impact on 5-year-survival. Accordingly, three of the four pN0 patients had a Ki67-index higher than the proposed cut-off but small tumor dimensions.

As far as local recurrence is concerned, all three patients had smaller tumors but safety margins between 8 and 10 mm and a Ki67-index higher than 85%. This could be an indication that a cut-off of 77.5%, as recommended here, may be a prognostic factor for a more difficult local control of the disease especially if the safety margins are not larger than 1 cm, which was supported by other studies, too. On the other hand, there were four patients (patients 1,2,3,4 and 14) with a Ki67-index of 90% although three of them (patients 2, 3 and 14) had a minimum safety margin lower than 10 mm did not show any recurrence. The average resection margins in our cohort were 12.8 mm for the patients without recurrence and 9 mm for the three patients with a local recurrence implying that at least 1 cm safety margins should be pursued.

Immunohistopathological markers are vital for diagnostics in MCC. In our study all patients expressed chromogranin A and CK20, 50% expressed synaptophysin and 37.5% CD56. Other authors also regarded CK20 as sensitive and specific marker for MCC with a specifity of 95% [31, 32]. Llombart et al. 2005 analysed 20 histopathological specimens of MCC and came to the conclusion, that Ki67 proliferation index, chromogranin A, synaptophysin and CK20 are ideal for the histopathological diagnosis of MCC [33]. This agrees with our data.

Limitations of this study, are its retrospective design and mainly the low number of patients, that does not allow a statistical multivariate analysis but only a qualitative examination of the data. Of course Kaplan-Meier analysis with such a low number of patients needs to be considered critically. The time span (2002–2021) that this study highlights is very long. One patient received 4 cycles of etoposide, Taxol, Carboplatin as adjuvant therapy. This occurred in 2012, at a time when immunotherapy with checkpoint inhibitors was not yet established in MCC.

## Conclusion

A Ki67- index of 77.5% stratified the patients in our cohort most efficiently. The Ki67-index could be used as prognostic parameter in MCC and could be important in clinical practice. A safety margin higher than 1 cm should be aimed. Immunotherapy could provide a long lasting remission in patients with distant metastases.

## Data Availability

Not applicable.
